# DRAMP 2.0, an updated data repository of antimicrobial peptides

**DOI:** 10.1038/s41597-019-0154-y

**Published:** 2019-08-13

**Authors:** Xinyue Kang, Fanyi Dong, Cheng Shi, Shicai Liu, Jian Sun, Jiaxin Chen, Haiqi Li, Hanmei Xu, Xingzhen Lao, Heng Zheng

**Affiliations:** 1 School of Life Science and Technology, China Pharmaceutical University, Nanjing, 211100 P.R. China; 2 State Key Laboratory of Bioelectronics, Southeast University, Nanjing, 210096 P.R. China; 3 School of Pharmaceutical Sciences, Wenzhou Medical University, Wenzhou, 325000 P.R. China; 4 The Engineering Research Center of Peptide Drug Discovery and Development, China Pharmaceutical University, Nanjing, 211100 P.R. China

**Keywords:** Protein databases, Antimicrobial resistance

## Abstract

Data Repository of Antimicrobial Peptides (DRAMP, http://dramp.cpu-bioinfor.org/) is an open-access comprehensive database containing general, patent and clinical antimicrobial peptides (AMPs). Currently DRAMP has been updated to version 2.0, it contains a total of 19,899 entries (newly added 2,550 entries), including 5,084 general entries, 14,739 patent entries, and 76 clinical entries. The update covers new entries, structures, annotations, classifications and downloads. Compared with APD and CAMP, DRAMP contains 14,040 (70.56% in DRAMP) non-overlapping sequences. In order to facilitate users to trace original references, PubMed_ID of references have been contained in activity information. The data of DRAMP can be downloaded by dataset and activity, and the website source code is also available on dedicatedly designed download webpage. Although thousands of AMPs have been reported, only a few parts have entered clinical stage. In the paper, we described several AMPs in clinical trials, including their properties, indications and clinicaltrials.gov identifiers. Finally, we provide the applications of DRAMP in the development of AMPs.

## Introduction

Infections caused by antimicrobial-resistant (AMR) bacteria have become a serious problem to global healthcare^1^. It is low estimates that at least 700,000 people die from AMR infections each year^2^. The emergence and worldwide spread of multiple-resistant “superbugs” cause an urgent need of novel antimicrobial medicine. A prospective weapon to fight against antimicrobial-resistant infections is antimicrobial peptides (AMPs). AMPs are a class of small cationic peptides, usually within 100 amino acid residues, produced by a variety of organisms, including bacteria, archaea, protists, fungi, animals and plants, and have broad-spectrum antimicrobial activity, immune regulation, wound healing, mediating apoptosis^3–6^. The first reported AMP, melittin, was isolated from bee venoms by Habermann *et al*. in 1952^7^. After that, a large number of natural AMPs have been reported, and these peptides were considered to be important components of their host defense system^8,9^. To date, more than 4,000 natural AMPs have been discovered^10–13^.

The progress of genomics and proteomics accelerates the discovery of novel AMPs, results the establishment of many AMP databases to store and annotate a wealth of AMP information including sequences, structures, activities, etc. These annotations can reveal the distribution, evolution and properties of AMPs and promote the development of AMPs as drug candidates^14,15^. Each AMP database has its special advantages due to differences in annotations and criteria for collecting data, such as some general databases: APD^16^ focusing on collecting natural AMPs and CAMP^17^ concentrating on classification of AMPs family characteristics, and some specific databases: Defensins Knowledgebase^18^, antiviral peptide database AVPdb^19^, and antiparasitic peptide database ParaPep^20^.

We have previously published the data repository of antimicrobial peptides (DRAMP 1.0) in April 2016. It is a manually annotated and open-access database of AMPs, dedicating to collecting natural, synthetic, patent, and clinical AMPs, and providing general, physical, structural, comment, and literature information^13^. Substantial AMPs discovered in recent years promote frequent updates of DRAMP. Specifically, compared with the first version, DRAMP 2.0 has increased more than 2,500 entries from 17,349 entries. With an eye to hemolysis of AMPs, hemolytic activity has been added, including detailed test data and corresponding red blood cells used in hemolytic assay. In addition, we have added some annotations, structures and download links of all data. Here, we report the updates and applications of DRAMP 2.0 and briefly introduced several clinically promising AMPs collected in DRAMP, which may serve as examples for data mining of DRAMP.

## Results

### DRAMP update

#### Entries of newly reported AMPs

**Fig. 1:**
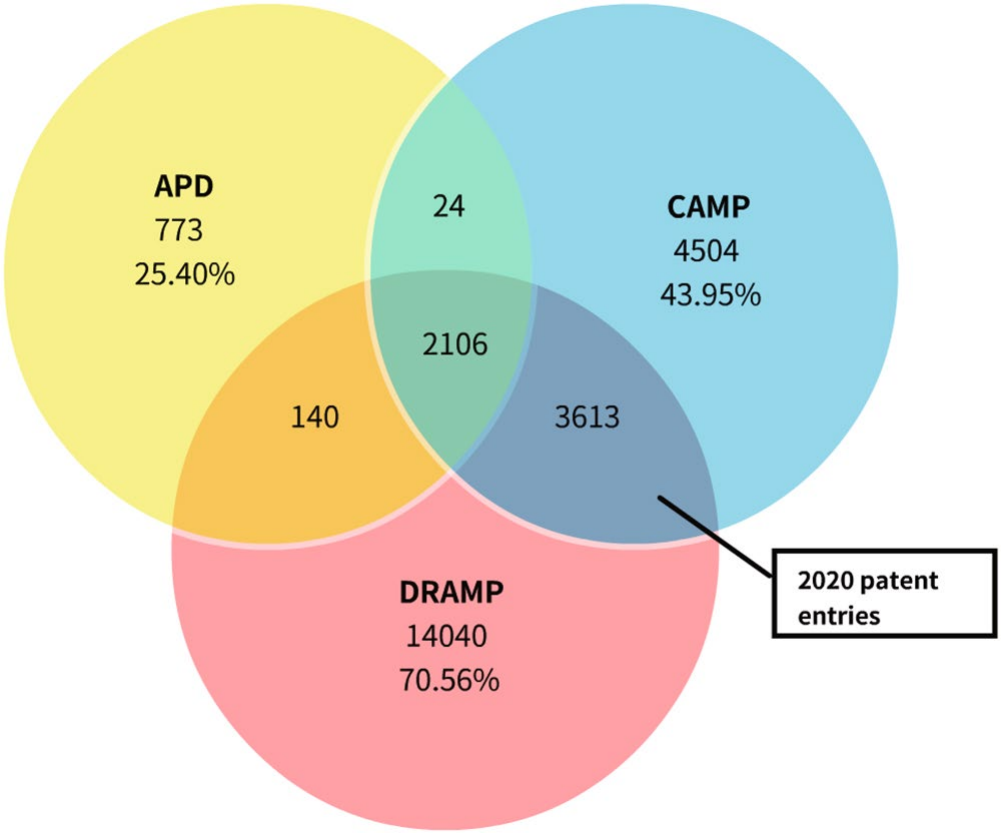
The number of overlapping and non-overlapping sequences of DRAMP, APD and CAMP. The number of non-overlapping sequences is calculated as a percentage in the corresponding database (data as of 2019.1.1).

Compared with the previous version, DRAMP 2.0 has added 2,550 new entries, including 513 general entries (340 natural entries and 173 synthetic entries), 2,035 patent entries and 2 clinical entries. Among them, 4 entries derived from archaea have been added. Most databases have their own unique sequences, as well as sequences that overlap with other databases. The overlapping sequences in DRAMP, APD and CAMP have been shown in Fig. 1. DRAMP has fewer overlaps with APD and CAMP, it contains 14,040 (70.56% in DRAMP) non-overlapping sequences. Both DRAMP and CAMP have established a patent dataset, there are 2,020 patent overlapping sequences covering 96.98% of CAMP patent dataset.

### New annotations

#### Hemolytic activity

**Fig. 2:**
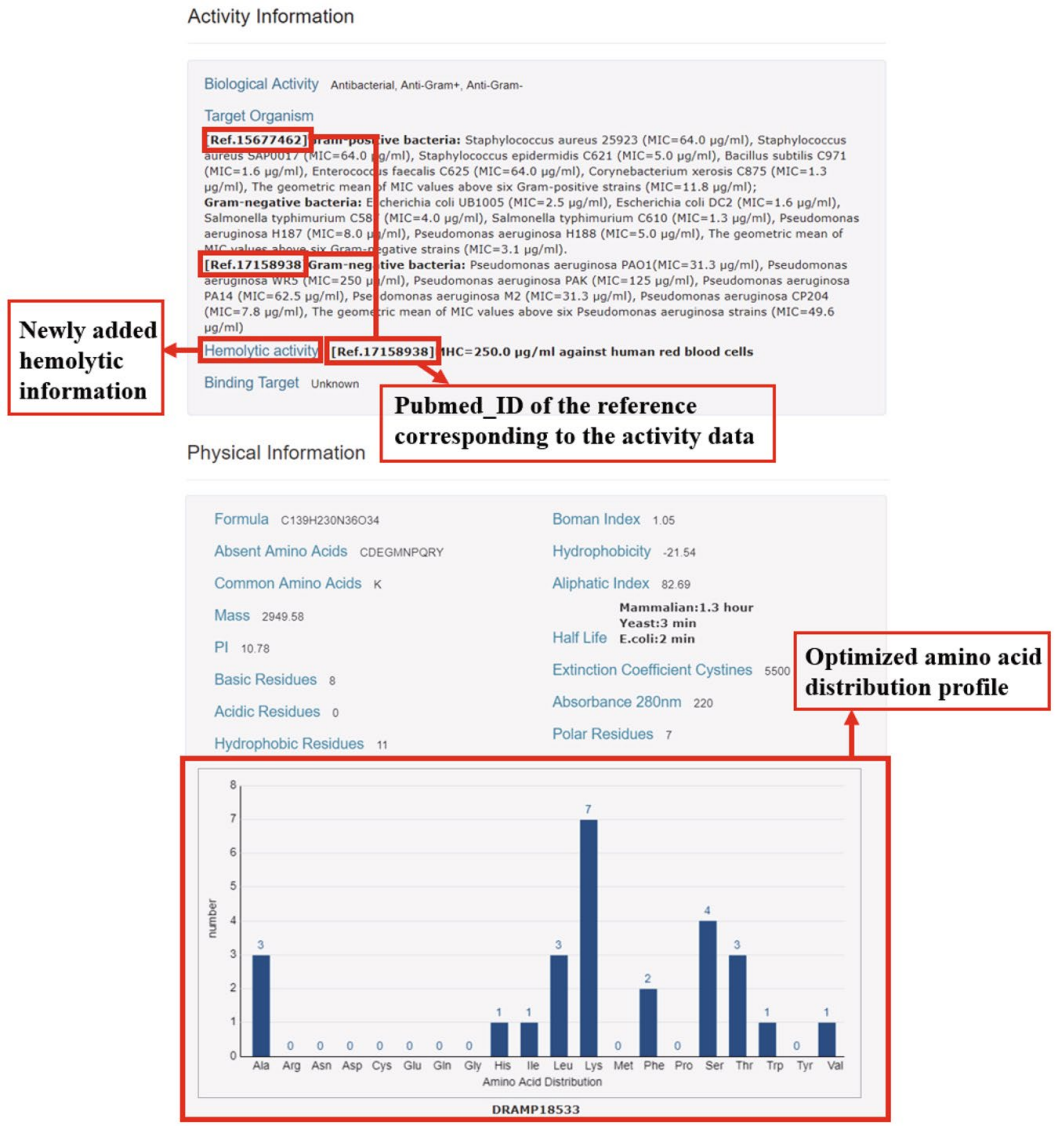
Visualization of detailed information page of DRAMP 2.0. In the red box is the update content. The screenshot in the figure is from DRAMP18533.

AMPs possess strong antibacterial activity, but they usually bring hemolytic side effect unfavorable for drug development. Reducing hemolytic activity is important for the development of AMPs as antimicrobial drugs^21^. CAMP^17^, LAMP^22^, SATPdb^23^ and Hemolytik^24^ are noted database embodying the hemolysis of AMPs, but only Hemolytik (Data is merged by SATPdb) contains hemolytic test data. Hemolytik (http://crdd.osdd.net/raghava/hemolytik/) is a specialized database containing experimental hemolytic information of ~3000 therapeutic peptides most of which are AMPs. It contains peptides’ names, sequences, hemolytic activities and putative structures, but unfortunately it does not contain antimicrobial test data of AMPs. All hemolysis information of Hemolytik have been hyperlinked to the SATPdb. In this update, we have added the hemolytic activities of AMPs. Hemolytic information contains PubMed_ID of relevant literature, hemolytic test results and corresponding red blood cells. An example of visualized information page is shown in Fig. 2. The detailed information of minimum inhibitory concentration (MIC) values, hemolytic activities and physical properties of AMPs may ease researchers to gather sufficient information and accelerate the development of novel low-toxic AMPs.

#### PubMed_ID for activity data

The authenticity of the data is the benchmark for database evaluation. As a manually curated open-access database, DRAMP tries to ensure the authenticity of the data. In the part of “Activity Information”, we have labeled the PubMed_ID of the corresponding reference for each activity data of AMPs. It can also help users to retrieve the relevant experimental methods, which are important when they compare different activity values.

#### Re-optimized the amino acid distribution profile

The amino acid distribution profile is quite useful for the prediction and design of peptides. To intuitively show the frequencies of the 20 natural amino acids, we re-optimized the amino acid distribution profile of each AMP in the part of “Physical Information”, allowing researchers to compare the differences and design rationally of AMPs, as shown in Fig. 2.

### New structures

The structure of the polypeptide determined by X-ray crystallography and NMR spectroscopy is usually embodied in the Protein Data Bank (PDB)^25^. Compared with the first version, we have updated 44 structures from the PDB.

**Fig. 3:**
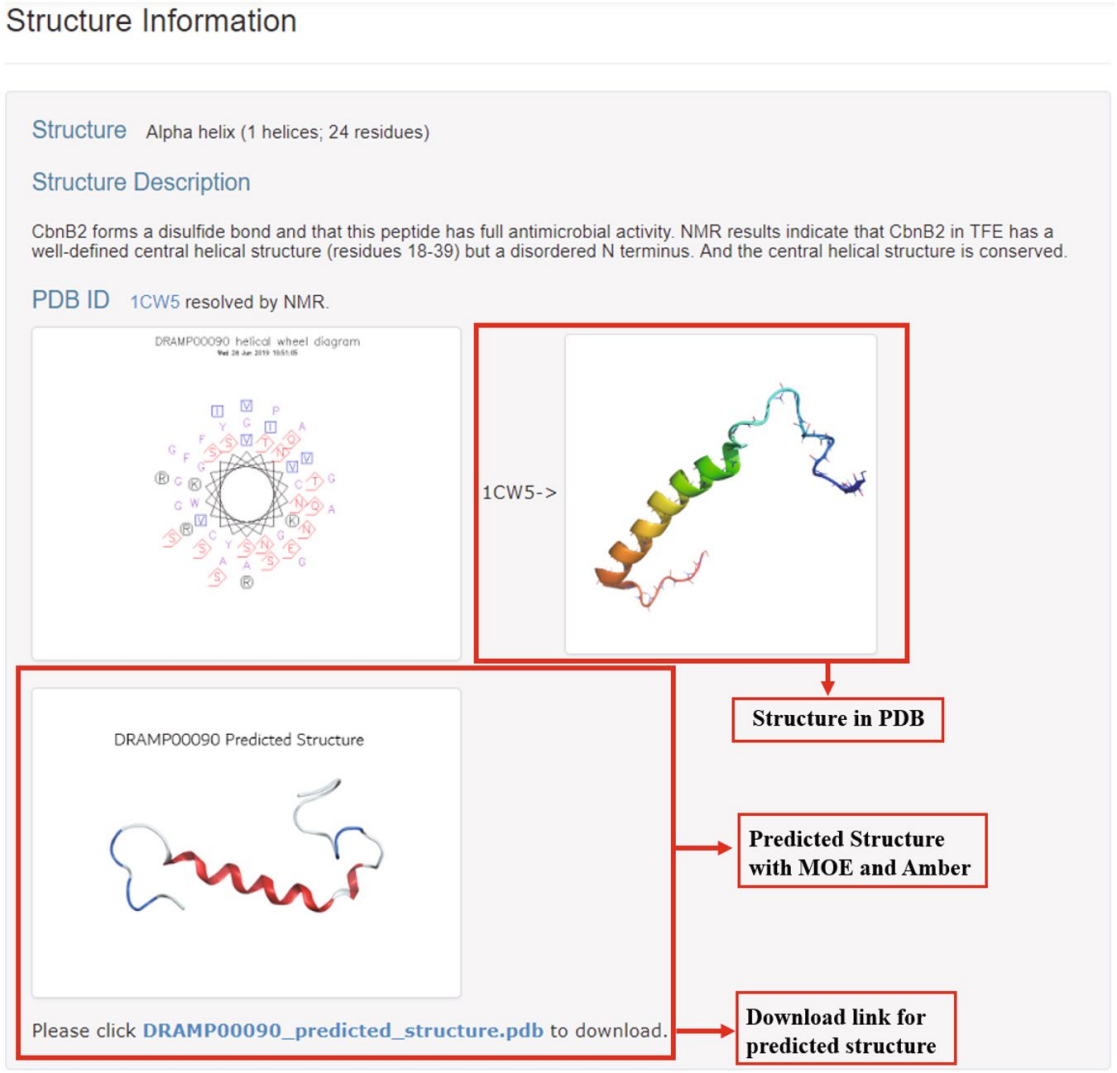
Visualization of detailed information page of DRAMP 2.0. In the red box is the update content. The screenshot in the figure is from DRAMP00090.

At present, the identified structural data is far from meeting the needs of researchers. In addition, due to the rapid development of computer technology, the newly identified sequences are exploding every year. Face this situation, structural prediction may be an appropriate way to bridge this gap^26–28^. Currently predicted structures or methods have been added to certain databases, such as: DBAASP^29^, AVPdb^19^, ParaPep^20^, SATPdb^23^. The 3D structure is generally predicted by homology modeling, which is mainly base on high homology between sequences^30^. Homology-modeled structures can be optimized by molecular dynamics and used to visualize the interaction between AMP and biomembrane^31–33^. In this paper, we used MOE2016 (https://www.chemcomp.com/) to establish an initial molecular structure by homology modeling, and Amber14 (http://ambermd.org/) to optimize the molecular structure by molecular dynamics, as previously reported^34^. In the second version of DRAMP, 82 predicted structures were added and these structures can be downloaded in pdb format (Fig. 3).

### Browse lists

**Fig. 4:**
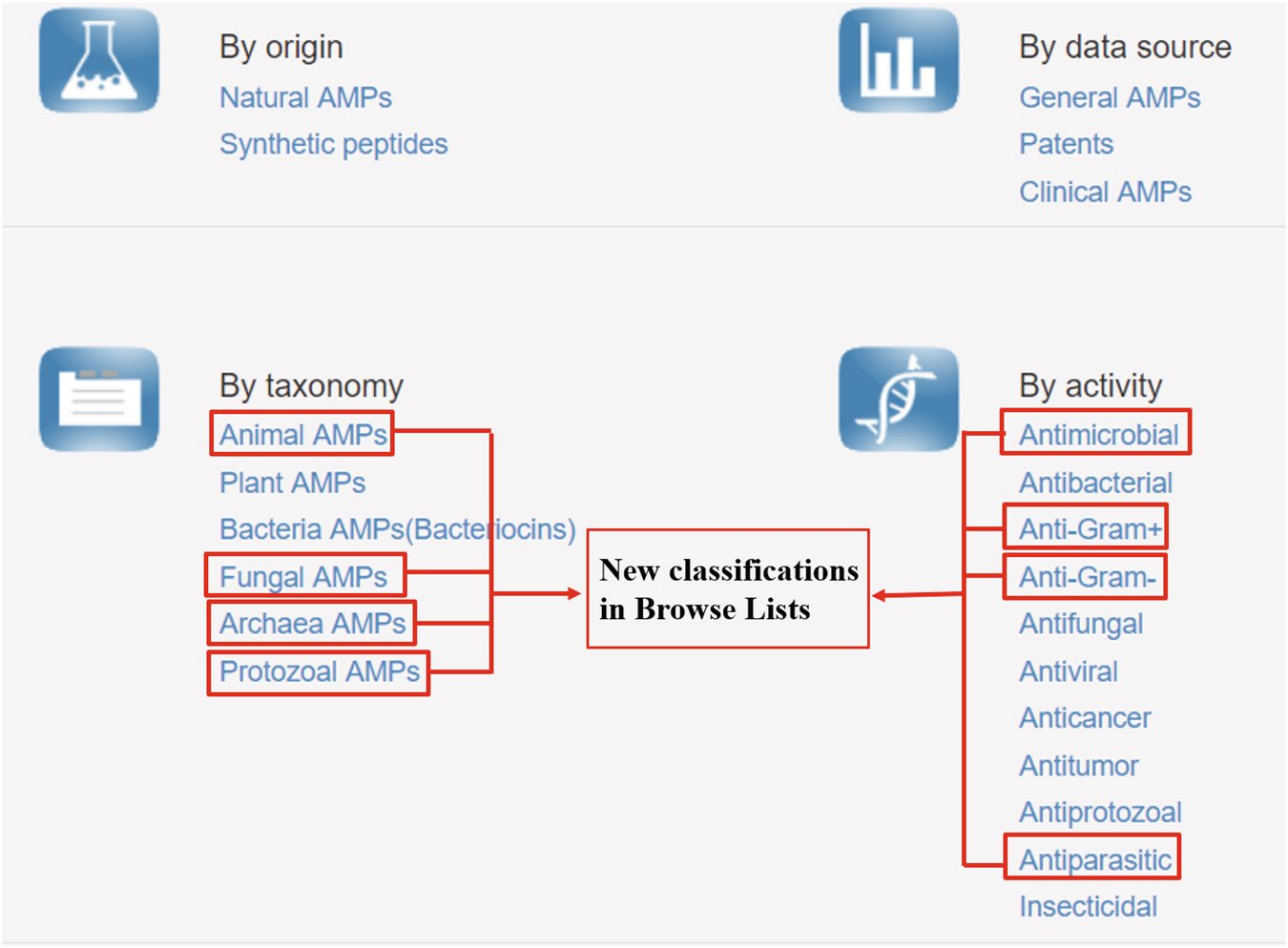
The new classifications by taxonomy and activity of DRAMP 2.0 in browse page (http://dramp.cpu-bioinfor.org/browse/). In the red box is the update content.

DRAMP classifies the data based on the data source, origin, taxonomy and activity in the browse page(http://dramp.cpu-bioinfor.org/browse/). In the DRAMP 2.0, natural data in general dataset can be further viewed by taxonomy. Compared with the first version, the natural AMPs can be linked to data which was classified in animal, plant, bacteria (included archaea), fungi and protists by taxonomy (Fig. 4).

**Fig. 5:**
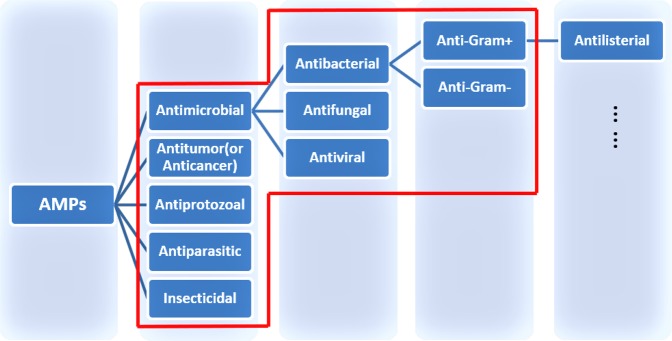
The relationship map of classifications by activity in DRAMP 2.0. In the red box is the main classifications of DRAMP.

In addition, DRAMP 1.0 was classified according to activities described in the literature, including antibacterial, antifungal, antiviral, anticancer, antitumor, antiprotozoal and insecticidal. The specific relationship map of activity is shown in Fig. 5. In this update, the activities of ‘Antimicrobial’, ‘Antiparasitic’, ‘Anti-Gram+’ and ‘Anti-Gram-’ have been added (Fig. 4). DRAMP 2.0 currently contains 11 searchable peptide activities which can be viewed via the browse page.

### Download links

We are delighted to share all the data and the website source code of DRAMP to advance the research and development of AMPs. We hope the open source distribution may promote the design of novel AMPs and encourage worldwide cooperation to face the coming AMR problem.

**Fig. 6:**
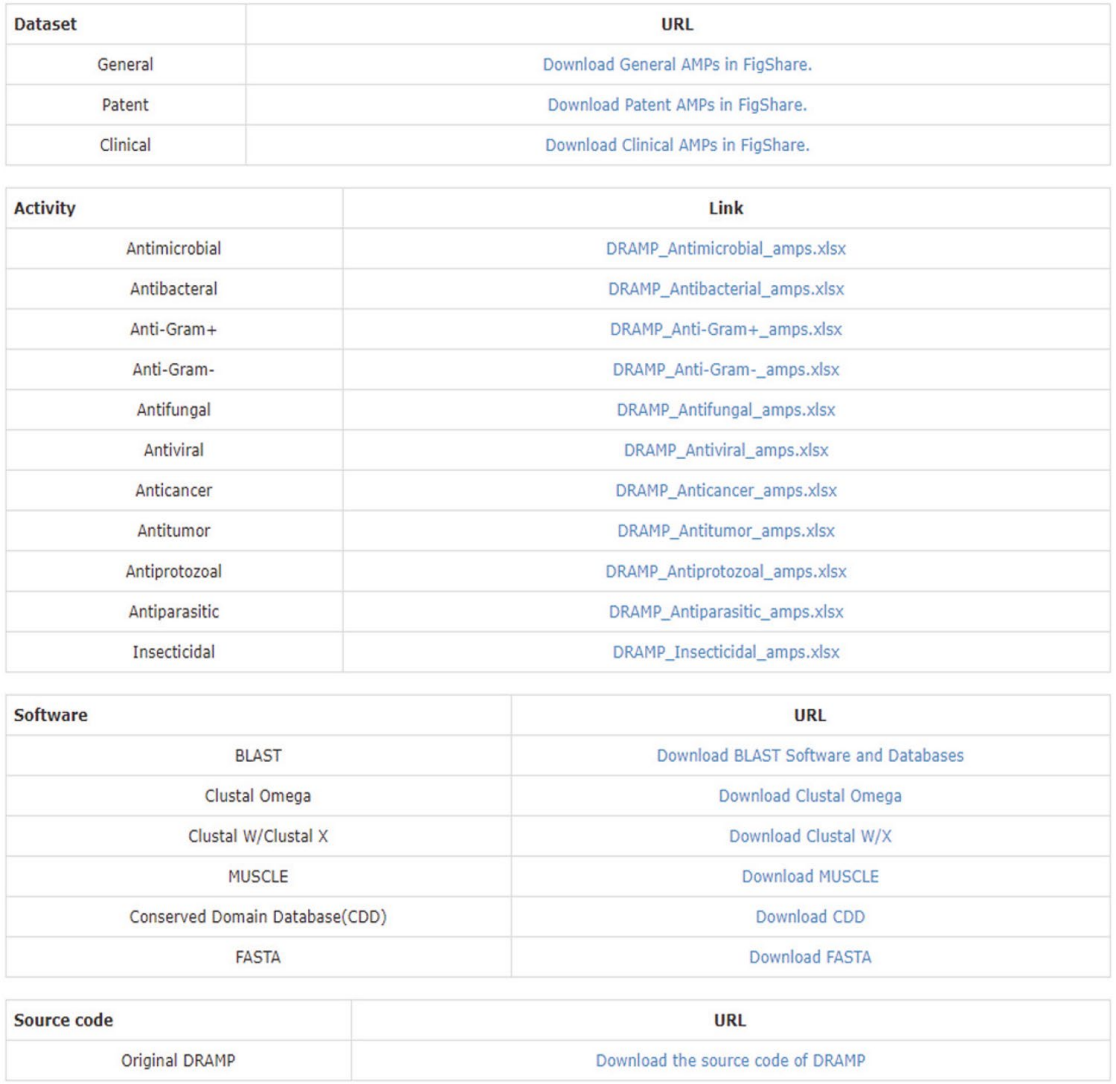
The new download page has been dedicatedly designed in DRAMP 2.0 (http://dramp.cpu-bioinfor.org/downloads/). The top-down four tables contain the links of datasets, activities, software, and website source code.

Therefore, we have completely updated the download page of DRAMP (Fig. 6). We divided the download page into 4 parts, which provide download of datasets, classified AMPs by activity in general dataset, the software used in DRAMP, and the original source code of DRAMP. All data contain complete annotations.

The first part provides download links for the three datasets of DRAMP, which have been deposited in figshare^35^. In the second part, we divided the data into 11 subgroups by activity (Figs 4 and 5). In the third part, we provided some frequently-used third-party bioinformatics tools which have been using in DRAMP to help researchers analyze AMPs easily. In the last part, there is a hyperlink to GitHub where DRAMP source code could be download for peer use. The open-source and easy-for-download property of DRAMP 2.0 may help researchers concentrate on data analysis instead of labor-consuming data collection, and accelerate the development of AMPs.

### Clinical AMPs

The clinical dataset in DRAMP embodies 76 entries. Many clinical trials of AMPs have failed even though they have so many attractive advantages. The safety of AMPs is currently the most vital and urgent problem to be solved^36^. Here, we briefly introduce some AMPs collected in our clinical dataset, which may serve as data mining examples to help users focus on most promising drug candidates of AMPs.

C16G2 (DRAMP_ID: 20760) is a synthetic AMP specifically for the dental caries pathogen *Streptococcus mutans*, developed by C3 Jian corporation^37^. C16G2 contains two connected domains by flexible tri-Gly linker region and the two domains are truncated forms of *Streptococcus mutans* competence stimulating peptide (CSP) pheromone and novispirin G10, respectively^38^. In the previous study, C16G2 has been found to effectively inhibit the growth of *Streptococcus mutan*^39^. Recently, numerous clinical studies on Phase II of C16G2 have been completed (NCT02594254, NCT02509845, NCT03196219, NCT02254993, NCT03052842, NCT03004365).

Omiganan (MBI-226/MX-226/CLS001, DRAMP_ID: 18160) is a synthetic derivative based on indolicidin that has been proved significantly to prevent local catheter site infections/colonization in patients undergoing central venous catheterization^32,40^. But a Phase III clinical trials (NCT00231153) carried out by Mallinckrodt showed lack of efficacy, the reason may be the natural L-Omiganan is instability and easy to be degradation by proteases. However, Ng *et al*.^41^ demonstrated that replacing all the amino acids of Omiganan with D-amino acids, the antibacterial activity was still remained, and the half-life of 80% of All-D-Omiganan degraded by protease increased from 10 min to more than 2 h. Currently, Omiganan is developed by Maruho corporation. Patient recruitment for atopic dermatitis (AD), usual type vulval intraepithelial neoplasia (uVIN), external genital warts, and acne vulgaris in Phase II has been completed (NCT02456480, NCT03091426, NCT03071679, NCT02596074, NCT02849262, NCT02571998) to assess the effectiveness of Omiganan^42^. To assess the safety of long-term administration for rosacea, a Phase III trial (NCT02576847) is completed, and Phase II pharmacodynamic studies for facial seborrheic dermatitis is recruiting patients (NCT03688971).

Murepavadin (POL7080, DRAMP_ID: 20774) is a 14-amino acid cyclic peptide for intravenous administration and belongs to the novel outer membrane protein targeted antibiotic (OMPTA)^43^. Murepavadin is obtained by amino acid substitution of protegrin I. In massive clinically extremely drug resistant (XDR) *Pseudomonas aeruginosa* isolates, even in some antibiotic-insensitive strains, Murepavadin exhibits potent activity, which is expected to be used to treat severe infections caused by *Pseudomonas aeruginosa*^44^. Murepavadin can target the lipopolysaccharide transporter D (LptD) on the outer membrane of bacteria to kill them^45^. Polyphor corporation is developing Murepavadin. There exist currently two clinical studies of Phase III (NCT03582007, NCT03409679) for nosocomial pneumonia and ventilator-associated bacterial pneumonia (VABP) respectively.

Dusquetide (SGX942, DRAMP_ID: 20773) is an analog of Innate Defense Regulators-1 (IDR-1) for the treatment of oral mucositis (OM) in patients being treated with concomitant chemoradiation for head and neck cancer^46,47^. According to the expected results, Phase III clinical trials (NCT03237325) is recruiting patients now.

### Application of DRAMP

DRAMP has been visited by more than 85,000 people since its opening, and cited by different research groups^48–50^.

Cipcigan *et al*.^51^ used known natural sequences whose lengths are 13 or 14 in DRAMP to mimic the insertion mechanism of AMPs. These AMPs have antibacterial activity against *staphylococcus aureus* and the MIC values were provided by DRAMP. The insertion depth of each AMP was calculated by molecular dynamics, and the average hydrophilicity of the AMPs was scored by GRAVY. It was found that the hydrophobicity of the AMPs was positively correlated with the insertion depth of the AMPs against *Staphylococcus aureus*. It means that the higher hydrophobicity is, the deeper AMPs insert into *Staphylococcus aureus* membrane. Therefore, it is pretty advantageous to flexibly use various AMP databases and bioinformatics tools to develop AMPs.

Nowadays in the field of data mining, machine learning has been receiving extensive attention with its powerful capabilities of data processing and application potential. In general, due to the limited 3D structures of experimentally determined AMPs, most AMP classifiers are based on 2D descriptors. Such as, CAMP_R3_^17,52^, ADAM^53^, and AntiBP2^54^ identify AMPs, BAGEL4^55^ and BACTIBASE^56^ mainly identify bacteriocins. We have developed a machine learning model based on 3D descriptors to predict AMPs and evaluate their antimicrobial effects by *in vitro* experiments^34^. The accuracy of the experimental optimal model in the training dataset is 92.59% and MCC is 0.84. The accuracy of this model in the independent test dataset is 100.00% and MCC is 1.00. We used this model to guide the rational design of 5 new AMPs, and the result of antibacterial assay was consistent with the predicted results^34^. This predictive classifier will be added to DRAMP to help researchers identify and rationally design AMPs.

## Conclusion

The research of AMPs is based on its broad-spectrum antibacterial, low resistance, immunomodulatory and antitumor advantages. Although some AMPs have entered clinical stage, there are still numerous obstacles to be solved, including: side effects (hemolysis, cytotoxicity, etc.), protease hydrolysis, sensitivity to salt or serum, high cost of large-scale production^4,57^.

To tackle the problems, DRAMP will provide more complete and detailed information about AMPs. In DRAMP 2.0, 5,084 General AMPs, 14,739 patent entries, and 76 clinical entries are embodied. There are 2,550 new entries added to the DRAMP compared with the previous version. We supplemented the hemolytic data and provided predicted structures for 82 entries, constructed by homology modelling and molecular dynamics^34^. These predicted structures have been used to design 3D descriptor-based AMP classifier by our lab, and the result of the antibacterial assay for the 5 designed AMPs confirmed that was consistent with the predicted results^34^. This model will become a part of the DRAMP prediction module. Furthermore, we have added a relationship map of classification by activity in the readme page (http://dramp.cpu-bioinfor.org/static/readme.php). Compared with the DRAMP 1.0, we have separated ‘Anti-Gram+’ and ‘Anti-Gram-’ from the antibacterial activity and added ‘Antimicrobial’, ‘Antiparasitic’ with them to the browse page.

More importantly, DRAMP 2.0 has shared all the data and annotations as well as the source code of the website with download links. We hope that the shared data will promote international cooperation, and accelerate the progress of antimicrobial research. The specific update list is shown in the Table 1.

**Table 1:** The update list of DRAMP 2.0.

Update classes	Contents
Entries	New General, Patent and Clinical AMPs
Source	Archaea
Activity	Separate Anti-Gram + and Anti-Gram- from Antibacterial activity
	Antimicrobial and Antiparasitic
Annotations	Hemolytic test data and corresponding red blood cells
	PubMed_ID corresponding to activity data
	Re-optimized amino acids distribution profile
Structures	New structures
	Predicted structures with MOE2016 and Amber14
	Download link for each predicted structure
Download page	Download URL for each dataset of DRAMP to figshare^35^
	Download link for each activity in Excel format
	Download URL for each software
	Download URL for DRAMP original source code to GitHub
Others	Statistics and Quicklink page updated

The clinically researched AMPs mentioned above show that AMPs are potential drug candidates for the treatment of various infectious diseases. However, numerous failed precedents indicate that many problems need to be overcome. In order to solve the problem that AMPs are easily hydrolyzed by protease, and to make AMPs more accurately target their expected sites, the modification of AMPs by the targeted nano-delivery system can decrease protease degradation or environmental influences^57^. Wang *et al*.^58^ discovered that polyionic complex (PIC) micelles formed by self-assembly of MSI-78 and the anionic copolymer methoxy poly(ethylene glycol)-b-poly(α-glutamic acid) (mPEG-b-PGlu) can be sustainably released *in vivo* to improve the stability, reduce hemolytic properties without affecting its antimicrobial activity. As for increasing antibacterial properties of AMPs, Godoy-Gallardo *et al*.^59^ immobilized hLF1-11 on five different titanium surface coatings, found that bacterial adhesion decreased and biomembrance formation was affected. The safety of AMPs is the biggest problem that hinders the passage of AMPs through clinical trials. Jin *et al*.^60^ found that hemolysis can be reduced if hydrophobic residues insert into the hydrophilic side of the helical structure. Ahmad *et al*.^61^ replaced L-Leu at ‘a’ and ‘d’ positions of the short leucine zipper peptide (SLZP) with D-Leu, which did not almost change in antimicrobial activity but significantly reduced hemolytic activity.

There exist many databases related to AMPs that are directly used by researchers. Not only do they integrate information on a great deal of AMPs, but also provide tools for predicting structures, activities, and toxicities of AMPs. This is also the future scenarios of DRAMP. DRAMP will continue to collect experimentally determined AMPs and re-update all existing data. DRAMP currently schedules to update at least every 3 months, providing more annotations, information and tools to facilitate researchers.

## Methods

### Data collection and update

The method of data collection and update is consistent with the first version of DRAMP^13^. The AMPs have the following characteristics: (1) less than 100 amino acids in length; (2) a clear mature sequence; (3) determined their activity. All information in the DRAMP is collected from PubMed, Uniprot, PDB and Lens by using keywords such as ‘antimicrobial peptide’, ‘antibacterial peptide’, ‘antifungal peptide’, or ‘hemolytic’. DRAMP contains a variety of detailed information of AMPs. In the first version, hemolysis is only annotated in the part of “Comments Information”. The newly hemolytic activities are obtained from literatures with experimental results of hemolytic tests and then added into DRAMP manually. These new annotations include corresponding red blood cells and activity of AMPs for hemolytic tests. DRAMP collects all activity test data as much as possible from papers. In order to prevent the possible errors, we have added literature sources for each data. All sequences and updates are available on the DRAMP website.

DRAMP was built on Apache web server (version 2.2.22) with Linux operating system. HTML, PHP and JavaScript were applied to develop the web interfaces as the front-end. MySQL server (version 5.5.29) was applied to manage the data as the back-end. The original source code of DRAMP website has been shared in GitHub. We have regular updates, backup, recovery and web optimization for DRAMP.

### Structure data collection and prediction

DRAMP in the first version included the AMP structures that were determined and registered in PDB. However, due to the small number of crystal structures determined in experiment, we used a reasonable method to construct 3D structures of AMPs^34^. We used MOE2016 (https://www.chemcomp.com/) to generate the initial structures for AMPs that do not have a crystal structure, and GB/VI as the scoring standard. Amber14 (http://ambermd.org/) with the FF14SB force field was used for molecular dynamics to optimize the structures. A truncated octahedral box was created and the system was solvated using TIP3P water before molecular dynamics. After optimization of system, the temperature was kept at 300 K for 100 ns and one conformation was reserved every 100 ps. The time step was 2 fs for all simulations.

In order to verify the accuracy of the predicted results, we performed the same prediction for the molecules which have their crystal structures. The predicted structures are similar to the experimental determined crystal structures, and the RMSD value is less than 2 Å. Although the predicted structures by the method are reliable, the time of molecular dynamics is pretty long. At present, we have predicted the structures of 82 AMPs (79 of which have no structure in PDB). The 82 predicted structures and download links in pdb format have been uploaded to the corresponding AMP structure information for public downloading and using. For the DRAMP_ID of 82 AMPs, please visit the update page (http://dramp.cpu-bioinfor.org/static/update.php). In order to prevent confusion between the crystal and the predicted structure, the pdb format files and the pictures of the predicted structures were labeled. We will continue to update the predicted structures of AMPs in the future.

All the data and sequences which are sorted by activity and dataset can be downloaded in Excel format from the DRAMP (http://dramp.cpu-bioinfor.org/downloads/). We have chosen the CC BY license and shared the data of DRAMP to researchers.

## Data Availability

The datasets analysed during the current study are available in the DRAMP database, http://dramp.cpu-bioinfor.org/downloads/, the APD database, http://aps.unmc.edu/AP/, and the CAMP database, http://www.camp.bicnirrh.res.in/. All data including general dataset, patent dataset and clinical dataset of DRAMP have been uploaded to figshare^35^, 10.6084/m9.figshare.c.4472759.

## Code Availability

The code of DRAMP original source and of preprocessing to re-produced the analysis results has been uploaded to GitHub: https://github.com/CPUDRAMP/DRAMP2.0.
